# Effectiveness of Riboflavin and Rose Bengal Photosensitizer Modified Adhesive Resin for Orthodontic Bonding

**DOI:** 10.3390/ph14010048

**Published:** 2021-01-10

**Authors:** Ali Alqerban

**Affiliations:** 1 Department of Preventive Dental Sciences, College of Dentistry, Dar al Uloom University, Riyadh 45142, Saudi Arabia; a.alqerban@dau.edu.sa; 2 Department of Preventive Dental Sciences, College of Dentistry, Prince Sattam Bin Abdulaziz University, Al-Kharj 11942, Saudi Arabia

**Keywords:** cross-linking agents, fluorescent dyes, oxidation reduction, photochemistry, photosensitizing agents, protein conformation, Rose Bengal

## Abstract

This study aimed to evaluate the effect of riboflavin (RF) and Rose Bengal (RB) photosensitizer modified adhesive resin on the degree of conversion (DC), and antimicrobial capacity after bonded to tooth surface. Different concentrations of RB and RF were prepared by homogenization method. An ultraviolet light source A (UVA) (375 nm wavelength, 3 mW/cm^2^ power) was used for 30 min irradiation. FTIR was performed for control and test adhesives to analyze the DC. Antibacterial testing was performed using the MTT assay. Metal brackets were bonded using the modified adhesives and subjected for SEM examination. The surfaces of teeth and metal brackets were examined at ×10 magnification for assessing adhesive remnant index (ARI) after PDT, 24 h and thermocycling. For DC, control group, 0.1% RB and RF after PDT showed the highest value. SEM imaging indicated lowest growth of *Streptococcus mutans* over 0.5% of RB-PDT and RF-PDT as compared to the control group. The MTT assay outcomes reported that the activity of *S. mutans* substantially decreased with the addition of a high amount of either RB or RF (*p* < 0.01). Mean ARI scores showed a significant difference between all groups. This study concluded that 0.1% of either RB or RF after PDT can be used for bonding orthodontic brackets to the tooth surface with substantial antibacterial properties.

## 1. Introduction

With the advancement of dentistry and patient knowledge, orthodontic therapy has gained popularity amongst the general population. The therapy is done to provide treatment for misaligned teeth that involves the use of orthodontic wires and brackets. The brackets used can either be metal or ceramic in origin [1,2]. Although orthodontic treatment assists in creating skeletal and dental improvement, this treatment modality is also responsible for creating an environment that favors bacterial growth [3,4,5]. 

The factors responsible for the increased development of bacterial biofilm include the application of orthodontic appliances close to the soft tissue structures, malaligned teeth, and improper oral hygiene maintenance [6]. The abundance of bacterial biofilm can lead to the development of oral health problems that include plaque-induced gingivitis, white spot lesions, and oral malodor (bad breath) [7,8,9,10]. The most common treatment modality used to tackle this problem addresses the repeated application of scalers (manual or ultrasonic). In adjunct to this, the patients are also advised to follow strict oral hygiene protocols [11].

To improve oral health care and patient satisfaction, dentists and scientists have been working together to establish treatment protocols that benefit on a larger scale. One such protocol that is being readily used in dentistry as an established therapeutic regimen is a photodynamic therapy (PDT) [12,13,14,15,16,17]. This treatment involves three main components, which include laser light (specific wavelength), nascent or free oxygen, and photosensitizer (PS). The procedure for this treatment is initiated by the application of laser light on the dye molecules. These dye molecules absorb the coherent light and transform a dormant singlet state to an excited triplet state. The excited molecules then react with the free molecular oxygen to form a highly toxic product called reactive oxygen species (ROS). These species further facilitate an irreversible oxidative movement in the bacterial cell composition, which ultimately leads to bacterial cell death [18,19,20]. Various types of photosensitizers are used to treat oral diseases including methylene blue, toluidine blue, indocyanine green, and many others. Rose Bengal (RB) and riboflavin (RF) have been extensively used for medical applications. RB and Rf both act as cross-linking agents. These photosensitizer-like agents act as inhibitors of collagen degradation, after the destructive activity of the acid-based bacteria, such as *Streptococcus mutans* (*S. mutans*) [21]. 

According to the authors’ knowledge, no study exists in the database that evaluated RB and RF as photosensitized proteins and modified inside the orthodontic adhesive resin. Therefore, this laboratory study aimed to characterize and evaluate the effect of RB and RF photosensitized orthodontic adhesive resin on degree of conversion, and antimicrobial capacity bonded to the tooth surface.

## 2. Results

**Figure 1:**
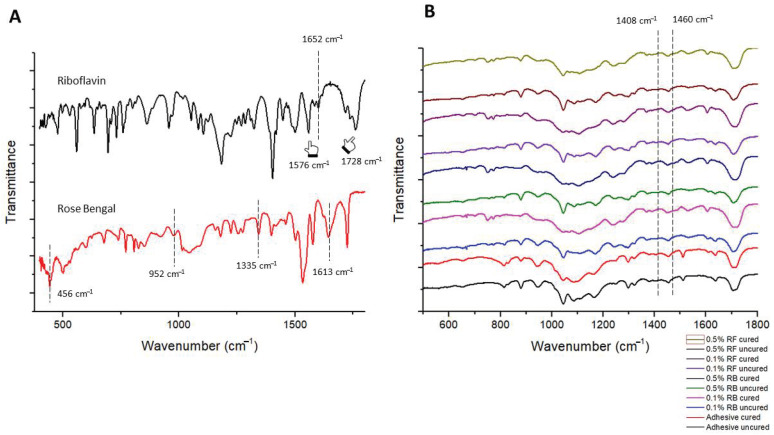
FTIR spectra of (**A**) riboflavin (black spectrum) and Rose Bengal (red spectrum) powder. Characteristic peaks at 1576 cm^−1^, 1652 cm^−1^, and 1728 cm^−1^ are noted. The spectrum at 1728 cm^−1^ indicates C=O stretching frequency of riboflavin. In the components, the aromatic C=C stretching mode of riboflavin appears at 1576 cm^−1^ and 1652 cm^−1^ (indicated with pointers). Rose-Bengal possesses important peaks at 456 cm^−1^ which indicates C–I bonding, at 952 cm^−1^ for C–C stretching vibration, and 1335 cm^−1^ and 1613 cm^−1^ for C=C stretching vibration and C=O stretching vibration, respectively. (**B**) After incorporating these powders in orthodontic adhesives, the ascribed peaks were evident forming strong bonds with the resin matrix.

The FTIR spectra of the powders riboflavin and rose Bengal are presented in Figure 1A. Characteristic peaks at 1576 cm^−1^, 1652 cm^−1^, and 1728 cm^−1^ are noted. The spectrum at 1728 cm^−1^ indicates C=O stretching frequency of riboflavin. In the components, the aromatic C=C stretching mode of riboflavin appears at 1576 cm^−1^ and 1652 cm^−1^ (indicated with pointers). Rose-Bengal possesses important peaks at 456 cm^−1^ which indicates C–I bonding, at 952 cm^−1^ for C–C stretching vibration, and 1335 cm^−1^ and 1613 cm^−1^ for C=C stretching vibration and C=O stretching vibration, respectively. After incorporating these powders in orthodontic adhesives, the ascribed peaks were evident forming strong bonds with the resin matrix. The degree of conversion was plotted for the area on the spectrum ranging between 1608 cm^−1^ to 1640 cm^−1^ (Figure 1B).

For DC, the groups control, 0.1% RB and 0.1% RF after PDT showed the highest degree of conversion indicating a significant conversion of monomer from the resin followed by lowest degree of conversion of 0.5% RB and 0.5% RF adhesive after PDT (Table 1).

**Figure 2:**
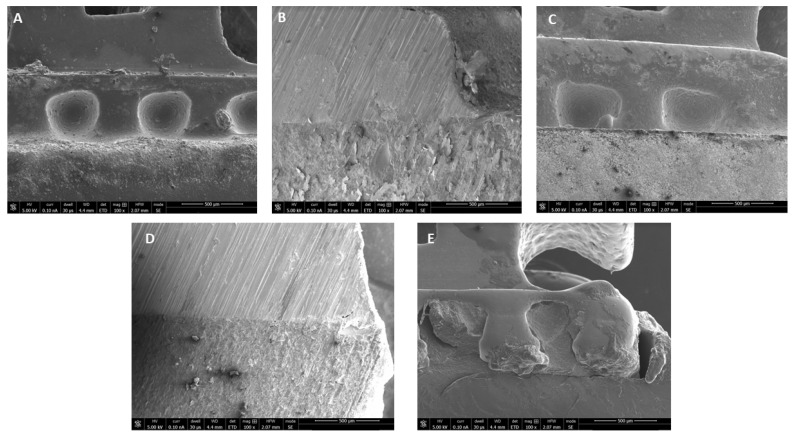
Representative SEM images of bracket-resin interface depicting excellent bonding of tooth surface with (**A**) Transbond XT (control); (**B**) 0.1% RB-PDT adhesive; (**C**) 0.1% RF-PDT adhesive; and (**D**) 0.5% RB-PDT adhesive; but (**E**) 0.5% RF-PDT adhesive, showed slightly poor bonding with defective adhesive within the mesh of the bracket.

The SEM images of the bonded brackets are shown in Figure 2A–E. It is noted that all adhesive showed a considerable excellent bonding between bracket mesh and tooth surface. However, 0.5% RF-PDT indicated slightly poor bonding with defective adhesive within the mesh of the bracket (Figure 2E).

**Figure 3:**
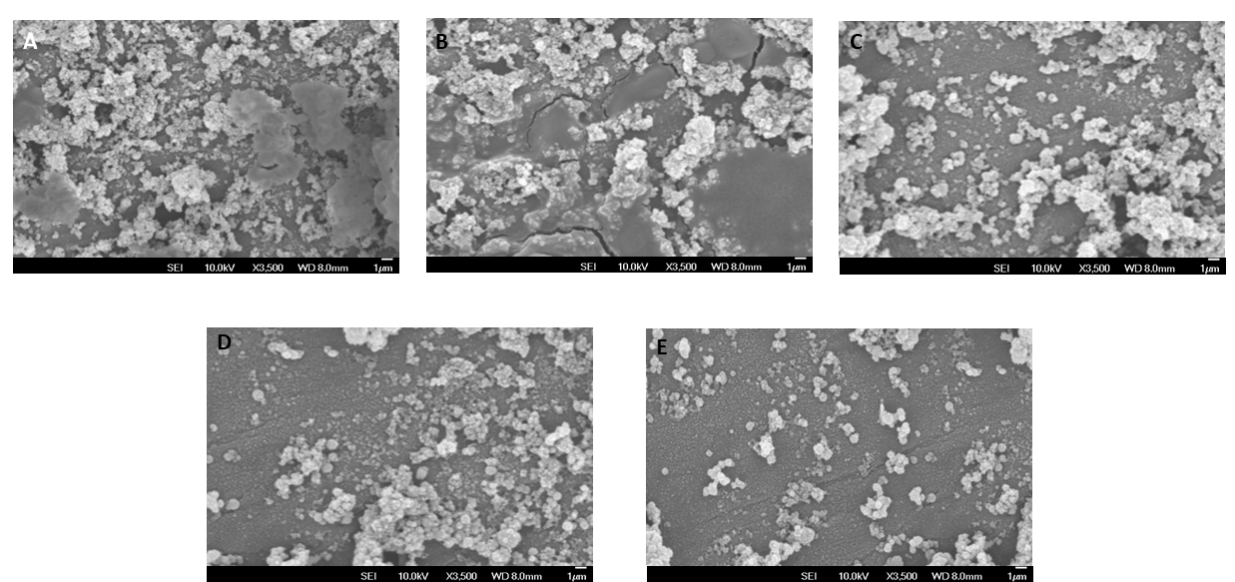
SEM micrographs showing growth of *Streptococcus mutans* over the surface of different modified adhesives used (**A**) Transbond XT (control); (**B**) 0.1% RB-PDT adhesive; (**C**) 0.1% RF-PDT adhesive showed low growth of *S. mutans*, Whereas (**D**) 0.5% RB-PDT adhesive and (**E**) 0.5% RF-PDT adhesive showed a substantial decrease in the microbial content of *S.mutans*.

SEM imaging of the experimental adhesives using 0.1% and 0.5% RB and RF indicated substantial low growth of *S. mutans* over 0.1% of RB-PDT and RF-PDT as compared to control group consisting of Transbond XT. Higher addition of RB and RF with 0.5% produced even lower growth of *S. mutans* over the modified adhesive surfaces (Figure 3A–E).

**Figure 4:**
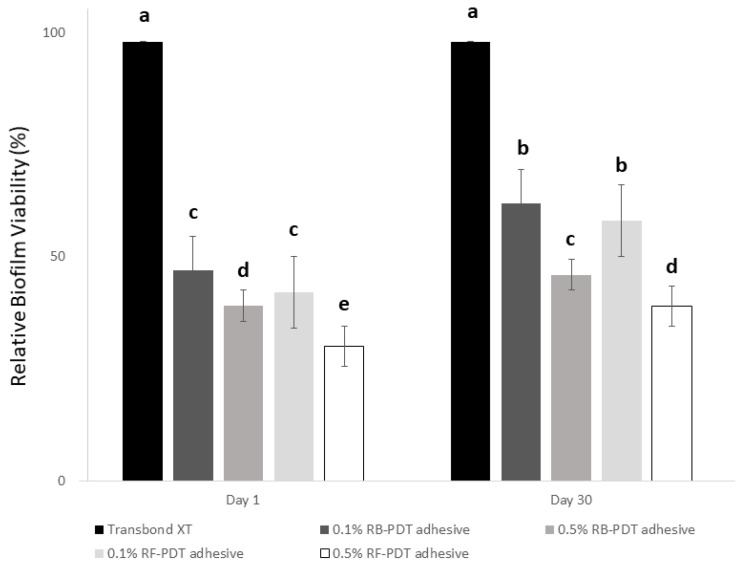
The relative biofilm viability expressed in percentage after Day 1 of incubation of *S. mutans* on experimental adhesives of 10 groups (5 for Day 1 groups, and 5 for Day 30 groups). The data are expressed as means and SD, groups with the dissimilar letters are statistically significant (*p* < 0.05).

The results of MTT assay are depicted in Figure 4. The outcomes showed that regardless of the day of assessment (Day 1 or Day 30), the metabolic activity of *S. mutans* substantially decreased with the addition of high amount of either RB or RF (*p* < 0.01). On comparison with day 1, although the metabolic activity of 0.1% RB or RF after PDT reduced to a certain extent after Day 30, 0.5% of either RB-PDT or RF-PDT showed relatively reduced viability of *S. mutans* well under 35%.

Table 2 depicts the results for ARI. The results of the ARI are shown in Table 2. When ARI was compared between groups in different time points (immediately after bonding, after 24 h bonding and after thermocycling), there was a significant difference in the mean ARI scores between all five groups.

## 3. Discussion

According to the data obtained after a comprehensive literature review, the present in-vitro experiment is said to be a novel study as it focused on orthodontic resin modification using RB and Rf with different concentrations (0.1% and 0.5%) after incorporating PDT to assess its effect on properties such as degree of conversion (DC), antimicrobial capacity and adhesive remnant index (ARI), respectively. According to the obtained results from the study, Group II and Group IV obtained better and equal results in comparison to the other included groups.

The resin–dentin interface is a very important interaction when it comes to restorative or orthodontic treatment. The durability of this interface comes into question when resistant oral bacterial influx usually harbors along with this complex interface. With increased acid accumulation, the adhesive–dentin interface is subjected to microleakage of bacterial by-products, further leading to secondary caries [22]. The secondary caries is instigated via the collagenase enzyme which is responsible for collagen degradation present in dentin [23]. During the procedure of bonding brackets, the conventional acid-etch technique also accounts for the production of an acidic environment on the tooth surface. This procedure allows the formation of micropores inside the tooth enamel by dissolving the calcium and phosphate ions [24]. So, to avoid further enamel degradation, the adhesive agent with antimicrobial properties is an absolute indication for tooth bonding. In our study, modified adhesives which included photosensitizing agents (RB, RF) were manufactured to test their effect in the presence of PDT. RB is a xanthene based fluorescent dye which has been used as a photosensitizing agent in numerous studies [25]. Being photoactive, this dye has been used to explore anti-microbial activity. Moreover, it has been used as a diagnostic tool in cancer and corneal surface defects. RF is a redox biological growth factor that belongs to the flavin group. Being a photosensitizer, it undergoes photoreactions at the nuclear levels to sensitize tumor cell destruction [26]. It has also been used for antimicrobial, antiviral and blood sensitizing applications. Furthermore, RF serves as a photoactivated cross-linking agent in corneal stiffness. Both the photosensitizers achieve intersystem conversion with the help of photo-illumination [27,28,29]. To achieve this, PDT was employed. PDT is based on a biochemical process that involves the use of a dye molecule in the presence of laser light of a specific wavelength and free oxygen. The exposure of laser light on the dye molecules instigates the process of conversion. This conversion is achieved by activation of dye molecules from a singlet dormant state to an excited triplet state. The excited triplet state reacts with the nascent oxygen to form reactive oxygen species (ROS). Being highly toxic, these free radical molecules play a significant role in the process of bacterial cell death [30,31,32]. According to previous studies, both RB and Rf are termed as photosensitive agents with excellent antimicrobial properties [33,34,35].

Regarding the aspect of tooth protein denaturation after thermal cycling, recent studies suggested that the photosensitizers RB and RF produce no effects. This can be supported by the fact that both photosensitizers differ in cross-linking procedures, as RF freely diffuses whereas, RB adheres tightly to the collagen matrix [36,37]. 

The degree of conversion (DC) is of great importance as it governs the physical and mechanical properties of the adhesive resins. It is also directly related to the conversion of monomers during the polymerization process [38]. According to the results of the present study, the DC is more prominent in control groups (0.1% RB-PDT and 0.1% RF-PDT) as compared to the test groups (0.5% RB-PDT and 0.5% RF-PDT). According to Table 1, a decrease in the levels of DC is observed after the increase in the concentration of both photosensitizers. A possible explanation for this finding could be because of the agglomeration of both the photosensitizers (RB, RF) used which could impede the curing process [39].

SEM images have been described in Figure 2 and Figure 3 In Figure 2, the images obtained depict that a strong bond was achieved between the enamel surface of the tooth and mesh of applied brackets. However, the use of 0.5% RF-PDT displayed relatively poor bonding in comparison to the other modified adhesive resins being used. The poor bonding observed might be due to the increase in the composition of riboflavin in the modified adhesive, which may have provided a shielding effect during the curing process of the bracket on the enamel surface [40]. For Figure 3, the use of RB-PDT and RF-PDT (0.5% concentration) produced a significant reduction in the *S. mutans* count. It has been reported in studies that RF and RB undergo conversion under the effect of photo-illumination by initiating a redox reaction which in turn causes changes in the bacterial cell contents, leading to necrosis [41].

Few limitations were observed for the present study. In this research, the use of Transmission electron microscope to study the sections after preparing photosensitizers mixed with orthodontic resins would have provided more accurate blending of the materials within resin matrix. Moreover, the translation of this in-vitro study into clinical experiments with the photodynamic system inside the oral cavity could result in different outcomes. Therefore, such studies are warranted in future. In addition, the use of a single type of bracket i.e., metallic is a limitation that can be addressed in other trials. The use of ceramic brackets alongside the modified adhesives could have provided data that would have been useful in devising better treatment protocol options. A reduced sample size could be another limitation for this in-vitro study. Future studies should consider more samples and compare this system within different types of ceramics surfaces and types of teeth other than molars.

## 4. Materials and Methods 

### 4.1. Materials and Chemicals

The materials used for the present in-vitro study included riboflavin and Rose Bengal and MTT assay kit ordered from Sigma Aldrich (St. Louis, MO, USA). The metallic brackets (0.022 × 0.028-in and 0.022 × 0.030-in) and orthodontic adhesive (Transbond XT) was ordered from 3M, Unitek (St. Paul, MN, USA).

### 4.2. Specimen Preparation

This experimental study was performed on 60 extracted human molar teeth which were acquired from patients who underwent extraction. The extracted teeth were thoroughly cleaned and rinsed with saline and later stored in 0.5% chloramine T solution at 4 °C until further use. Before establishing the criterion for inclusion and exclusion, all the teeth were examined thoroughly with the naked eye and under a stereomicroscope with ×10 magnification. Extracted molar teeth were visually inspected for any fractures, enamel loss, or caries.

### 4.3. Photosensitizer-Modified Experimental Orthodontic Adhesives

The 0.1 and 0.5 wt.% RB and RF modified adhesives were prepared by homogenization method, respectively. A total of 5 mg and 25 mg of the powders (RB and RF) were added in 5 mL of orthodontic resin (Transbond XT, 3M, Unitek, St. Paul, MN, USA), respectively [21]. The mixtures were given a thorough mixing in an ultra-homogenizer sonicator (SALD 2300 Shimadzu, Shimadzu Corporation, Kyoto, Japan) for 10 min. These prepared solutions were kept in the dark to avoid any occurrence of photobleaching. 

### 4.4. Groups

The experimental groups were divided based on the orthodontic adhesive modification. The groups were divided into following 5 groups, Group I: Transbond XT (control), Group II: 0.1% RB–PDT adhesive, Group III: 0.1% RF–PDT adhesive, Group IV: 0.5% RB–PDT adhesive and Group V: 0.5% RF–PDT adhesive.

### 4.5. Photodynamic Therapy (PDT) Protocol

The PDT protocol for this particular research was done per the method described by Arboleda et al. [21]. The samples were fixed in a petri dish. The petri-dish was set 1 cm from the irradiation source. The total spot diameter was carefully checked to be at 8 mm. Ultraviolet light source A (UVA) of 375 nm wavelength was employed to irradiate the petri-dish. The value of irradiance was 3 mW/cm^2^. Moreover, the time of exposure was 30 min.

### 4.6. Degree of Conversion

Fourier-transformed infrared spectroscopy (FTIR) (ThermoFischer Scientific, Waltham, MA, USA) was performed for control and test adhesives to analyze the degree of conversion (DC). For the spectral analysis, the uncured adhesive specimen was exposed to oil-free air, whereas the cured adhesive specimen was constantly kept in contact with the sensor. The absorbance peaks for the uncured specimens were measured by using FTIR spectrometer (Nicolet 6700, ThermoFisher Scientific, Waltham, MA, USA). To measure the degree of conversion, the range of spectrum was set between 400–4000 cm^2^. The same analytical tests were used for the cured specimens after curing. A baseline technique was used to obtain the C=C absorbance peak/1638 cm^−1^ (unpolymerized methacrylate stretching vibration) and C–C reference peak at 1607 cm^−1^ (stretching vibration of the aromatic ring). The formula used to calculate the degree of conversion is as follows:

Degree of conversion = [1 − (C_aliphatic_/C_aromatic_)/(U_aliphatic_/U_aromatic_)] × 100% (1)

### 4.7. Anti-Bacterial Testing

The bacterial strain of *S. mutans* was grown for two days at a temperature of 37 °C in an anaerobic environment. A total of nine specimens using both the control and modified orthodontic resin specimens (0.1% RB–PDT adhesive, 0.5% RB–PDT adhesive, 0.1% RF–PDT adhesive and 0.5% RF–PDT adhesive) were used for antimicrobial testing. To obtain the inoculation medium, the prepared medium of BHI supplementing 1% (*w*/*v*) sucrose was diluted. Each adhesive disc was placed in each well of 48 well-plate. Each well plate was carefully smeared in 1 mL of inoculation medium, before being incubated for 2 days for growth of *S. mutans*, in an anaerobic environment. The temperature was set at 37 °C. The buffer solution was used to gently rinse the *S. mutans*-coated specimens.

The viability of *S. mutans* biofilm was tested using the MTT assay. Specimen from each beam was transferred to one well of 24-well plate containing 1 mL of 3-(4,5-Dimethyl-2 thiazolyl)-2,5-diphenyl-2*H*-tetrazolium bromide (MTT; Sigma-Aldrich, St. Louis, MO, USA) solution, and incubated at 37 °C for 3 h anaerobically. After that, the MTT solution of each well was washed out and replaced by 2 mL of DMSO to dissolve the formazan. This was followed by gently shaking for 15 min and the supernatant collected from each well was estimated at 600 nm using a spectrophotometer (ThermoFischer Scientific, Waltham, MA, USA) after 24 h and 30 days.

### 4.8. Placement of Brackets on Enamel Surface

Metal brackets (3M, Unitek, St. Paul, MN, USA; MBT, slot 0.022 × 0.028 inch) were used to be bonded on extracted molar teeth. Transbond XT light cure primer and adhesive modified with 0.1% and 0.5% RB–PDT adhesive and 0.1% and 0.5% RF–PDT adhesive along with the control adhesive were used to bond brackets by using the conventional etch-and-rinse protocol. The etchant gel (37% phosphoric acid) was applied for 30 s on all the enamel surfaces, after being rinsed with water and dried for 20 s each. All the selected teeth in each group were subjected to their respective resin application. The process of light-curing was applied for 20 s where mesial and distal sides were each subjected to 10 s, respectively.

### 4.9. Scanning Electron Microscopy (SEM)

A random sample from each of the study groups was selected for the studying resin-bracket interface and bacterial culture over the discs under a scanning electron microscope (FEI, Tokyo, Japan). The process of fixation of these specimens was done by using a combination of 4% paraformaldehyde and 2% glutaraldehyde in 0.1 M sodium cacodylate (NaCac) buffer solution. The pH for these samples was maintained at 7.4 and was allowed to be stored overnight. The specimens were then subjected to 2% osmium tetraoxide before undergoing dehydration via ethanol (50 to 100%). These dried discs were then taped with double-sided copper tape. 10 kV disc images were obtained with the help of a through-lens detector (TLD) for secondary electron imaging. 

### 4.10. Adhesive Remnant Index (ARI) Assessment

After the process of enamel debonding, the surfaces of teeth and metal brackets were examined with the help of a stereomicroscope (EMZ-TR, MEIJI, Saitama, Japan) at ×10 magnification. The ARI scoring system was employed to score the remnants of adhesive resin. The scoring system was graded as:

0—total absence of resin on the surface enamel, 1—<50% of resin remaining on the surface enamel, 2—>50% of resin remaining on the surface enamel, 3—the complete presence of resin on the surface enamel having a complete impression of the base of metallic brackets. ARI was estimated immediately after PDT, after 24 h and after thermocycling process.

### 4.11. Statistical Analysis

The statistical analysis for the obtained data was executed by using the SPSS software (Version 26, SPSS Inc., IBM, Armonk, NY, USA). The data was reported in mean and standard deviation (mean ± SD). The value of statistical significance for each test was set at *p* < 0.05. The one-way ANOVA with Student–Neumann–Keuls post hoc test for the assessment of differences in the degree of conversion. For the MTT assay analysis, the Friedman test with post-hoc Tukey’s Kramer test was performed, whereas the Kruskal–Wallis test was employed for analyzing the ARI scores.

## 5. Conclusions

This study showed that a versatile RB or riboflavin modified orthodontic adhesive was developed, and the impact of addition of RB and RF after photoillumination on antimicrobial capacity and orthodontic bonding characteristics were studied. Our study suggested that 0.1% of either RB or RF after PDT can be used for bonding orthodontic brackets to the tooth surface with substantial antibacterial properties. Increased concentration of RF (0.5%) produced better antimicrobial results, but relatively poor bonding properties. 

## Figures and Tables

**Table 1:** Degree of conversion in percentage of all groups.

Groups	Degree of Conversion (%Mean ± SD)	Tukey (*p* < 0.05)
Transbond XT	*n* = 48.5 ± 6.6	A
0.1% RB-PDT adhesive	*n* = 46.8 ± 7.3	A
0.1% RF-PDT adhesive	*n* = 46.2 ± 5.8	A
0.5% RB-PDT adhesive	*n* = 41.3 ± 4.3	B
0.5% RF-PDT adhesive	*n* = 39.7 ± 5.1	B

**Table 2:** Mean adhesive remnant index (ARI) scores for all groups immediately after photodynamic therapy (PDT), after 24 h and after thermocycling.

Groups (Time Point)	Mean ARI	*p*-Value
**Transbond XT** *Immediately after* *After 24 h* *After thermocycling*	*n* = 1.33 ± 0.57 *n* = 1.27 ± 0.69 *n* = 1.24 ± 0.71	*n* = 0.022
**0.1% RB-PDT adhesive** *Immediately after* *After 24 h* *After thermocycling*	*n* = 1.47 ± 0.65 *n* = 1.42 ± 0.71 *n* = 1.37 ± 0.75	
**0.1% RF-PDT adhesive** *Immediately after* *After 24 h* *After thermocycling*	*n* = 1.72 ± 0.55 *n* = 1.68 ± 0.46 *n* = 1.61 ± 0.84	
**0.5% RB-PDT adhesive** *Immediately after* *After 24 h* *After thermocycling*	*n* = 1.91 ± 0.85 *n* = 1.73 ± 0.77 *n* = 1.66 ± 0.90	
**0.5% RF-PDT adhesive** *Immediately after* *After 24 h* *After thermocycling*	*n* = 1.66 ± 0.83 *n* = 1.62 ± 0.97 *n* = 1.58 ± 0.74	

## Data Availability

Not applicable.
